# A Comparative Analysis of Honeypots on Different Cloud Platforms

**DOI:** 10.3390/s21072433

**Published:** 2021-04-01

**Authors:** Christopher Kelly, Nikolaos Pitropakis, Alexios Mylonas, Sean McKeown, William J. Buchanan

**Affiliations:** 1 School of Computing Edinburgh Napier University, Edinburgh EH10 5DT, UK; 40204337@live.napier.ac.uk (C.K.); S.McKeown@napier.ac.uk (S.M.); B.Buchanan@napier.ac.uk (W.J.B.); 2 Eight Bells LTD, Nicosia 2002, Cyprus; 3 Department of Computer Science, University of Hertfordshire, Hatfield AL10 9AB, UK

**Keywords:** cloud computing, cybersecurity, honeypot, Google Cloud, AWS, Microsoft Azure

## Abstract

In 2019, the majority of companies used at least one cloud computing service and it is expected that by the end of 2021, cloud data centres will process 94% of workloads. The financial and operational advantages of moving IT infrastructure to specialised cloud providers are clearly compelling. However, with such volumes of private and personal data being stored in cloud computing infrastructures, security concerns have risen. Motivated to monitor and analyze adversarial activities, we deploy multiple honeypots on the popular cloud providers, namely Amazon Web Services (AWS), Google Cloud Platform (GCP) and Microsoft Azure, and operate them in multiple regions. Logs were collected over a period of three weeks in May 2020 and then comparatively analysed, evaluated and visualised. Our work revealed heterogeneous attackers’ activity on each cloud provider, both when one considers the volume and origin of attacks, as well as the targeted services and vulnerabilities. Our results highlight the attempt of threat actors to abuse popular services, which were widely used during the COVID-19 pandemic for remote working, such as remote desktop sharing. Furthermore, the attacks seem to exit not only from countries that are commonly found to be the source of attacks, such as China, Russia and the United States, but also from uncommon ones such as Vietnam, India and Venezuela. Our results provide insights on the adversarial activity during our experiments, which can be used to inform the Situational Awareness operations of an organisation.

## 1. Introduction

It is estimated that one in four businesses will run their applications solely on the cloud within a year [1]. This involves moving all their IT infrastructure to cloud-based providers to utilise either a private and/or public cloud structure. As cloud providers’ IP address subnets are public knowledge, this has opened channels for attackers to deploy mass scanners to automate attacks and take advantage of poorly configured services and protocols that are deployed in cloud instances.

In the current threat landscape, threat actors are active constantly across the world, attempting to exploit new and existing vulnerabilities, which often could be decades old. Such an incident happened recently, where yet another large-scale breach was witnessed with Capital One’s customers data being exposed [2]. A misconfigured Amazon Web Services (AWS) instance allowed the exposure of hundreds of thousands of customers details, including bank account and social security numbers. It is therefore necessary to learn and investigate how cyber criminals scan and interact with systems that are accessible publicly. To understand such activity honeypots are often deployed, which can emulate a vast range of different services across a system and network to lure potential attackers into initiating interaction. The results gathered from honeypots can be extremely useful for organisations, security teams or cyber security research overall. This holds true as the analysis of the honeypot data can improve existing intrusion detection systems (IDS) and ensure that knowledge is kept current regarding the tactics techniques and procedures of threat actors in the current threat landscape.

Honeypots were designed as a deployable resource and are expected to undertake various attacks across the Internet, to be compromised and collect data [3]. In contrast to IDS, honeypots provide malicious actors access to a constrained environment with the intention of monitoring their actions in order to determine their motivations and end goals. This can then allow companies or security experts to learn from the attacks and modify, upgrade and improve the existing security infrastructure [4].

Honeypots can operate both internally and externally to lure in attackers. By operating internally they can collect data from an internal threat actor, such as insider threats [5,6]. They can also collect data from external threat actors by attracting them towards their open and poorly secured ports. External facing honeypots can allow organisations to see the kind of attacks that target them.

By collecting data such as threat types, session duration, login credentials and Common Vulnerability Exposure (CVE) numbers exploited, it allows security experts to advise organisations how they can improve their security posture [7]. Data can also be inspected to determine possible geolocations of attackers or the source IP addresses. This data can be used to constantly update firewalls to block known or suspicious IP addresses. Researchers could benefit from Honeypots by discovering elements of active attacks from the probing and reconnaissance, to the actual attack vectors once the exploit has been successful. As a result, the detection accuracy for active attacks can be improved but as the honeypot is contained in an isolated environment, the security of real assets is not impaired.

Our work investigated the different types of attacks being deployed across the Internet against the most popular Infrastructure as a Service (IaaS) cloud computing environments, namely Google Cloud Platform (GCP), Amazon Web Services (AWS) and Microsoft Azure. In doing so, by analysing and visualising the collected data, it sheds light on adversarial activities which were active while we conducted our analysis. Our results highlight the importance of use of publicly available threat intelligence information and indicators of compromise collected from honeypots, by following a similar methodology as in our work, in order to inform an organisation’s Situational Awareness operations. In summary, our work makes the following contributions:

- We provide an analysis of attackers’ activity on honeypots, which are deployed on the most popular cloud providers, namely AWS, Azure and Google Cloud. We analyse the volume of the attacks and extract the commonly targeted services, used credentials, exploited vulnerabilities, and combine them with threat intelligence feeds.
- We provide a region-based analysis of attackers’ activity by capturing and analysing data from honeypots operating into different regions, namely North America, Asia and Europe.

The rest of the paper is organised as follows: Section 2 provides background on cloud computing and honeypots as well as presents related work. Section 3 describes our methodology and Section 4 presents our results. Finally, Section 5 concludes the paper and discusses future work.

## 2. Background

### 2.1. Cloud Computing

The term cloud refers to the *floating* nature of the data, as it can move around between different physical servers and location [8]. The National Institute of Standards and Technology (NIST), defines three different cloud service models [9] as:

- Software as a Service (SaaS)—is used to describe applications that businesses subscribe to and run on cloud infrastructure over the Internet.
- Platform as a Service (PaaS)—the vendor provides higher-level application services where the business can create its own custom applications. This way the business can freely configure and deploy its application, however it is still controlled by the provider with its hardware and resources.
- Infrastructure as a Service (IaaS)—provides a backbone by providers such as Google, Amazon, and Microsoft where the services can be rented by businesses.

Irrespective of the various service models, a company may choose to either make use of external infrastructure or host their own cloud platform. To be considered a private cloud, the cloud service must not share its resources with any other organisation. A public cloud on the other hand, is utilised by multiple customers in which a cloud provided sells its cloud services. Elaborating on cloud service models, public clouds make use of SaaS, PaaS, and IaaS services. This way, customers access the services they are paying for, or renting, over the Internet from public cloud, which run on remote servers that the provider will manage. Opposed to the private cloud, a public cloud will share its services among several different customers, but in a way that each individual customer’s data and applications are kept private and not accessible from other customers sharing the services [10].

### 2.2. Honeypots

One of the first uses of a honeypot showed up in 1986 which involved a high-profile, well covered story. As described in their book The Cuckoo’s Egg [11], Clifford Stoll, a sysadmin for US Berkeley, discovered that somebody was accessing the system with superuser access. To attempt to catch this attacker, Stoll implemented two defences deployed in a honeypot form. This included attaching borrowed terminals to all the incoming phone lines on the system, then waiting for the unknown attacker to dial in. This approach allowed them to discover exactly what the attacker was looking for and he even managed to lure them into an entirely fictitious department he had created of a *Star Wars missile defence system*. This led to the arrest of a German working for the KGB and resulted in the first successful honeypot facilitation. A similar endeavour by Cheswick [12] in 1990 popularised the use of the term honeypot, inspiring the Honeynet Project [13] in 1999, which still remains active to this day.

Honeypots can be categorised based on their uses and purpose, dependent on the different services they provide and what interaction level they utilise. The 2 types of honeypots consist of Research and Production honeypots.

The primary principle of a research honeypot is to examine how malicious actors perform their attacks and see the diverse range of techniques they harness. Canner [14] explains it as a platform to watch the infiltration techniques used by attackers to access systems, escalate privileges and then explore their way around the system or network. Such honeypots are usually setup by academics, security researchers, government agencies and security companies in order to assess and measure the threat landscape. This activity provides data which can be analysed and used to discover the tactics, techniques and procedures of threat actors.

Production honeypots take a more specific, precisely configured infrastructure to mitigate current or potential attacks against an organisation [15]. They are deployed in close proximity to the organisations current production infrastructure and follow a similar security architecture. Such an approach allows the honeypot to appear almost identical to the company’s other services to lure an attacker to it, instead of the legitimate services. The emulated services are specifically configured, usually with similar operating systems and services on the standard ports. This allows data gathering to occur to examine what aspect of the infrastructure failed and why an attack was successful [16]. These honeypots may also be useful in distinguishing between automated attacks and manual, human-led, attacks [17], which will allow the creation of a realistic threat model.

Honeypots offer different levels of interaction, namely low, medium, and high, which reflects how much an attacker can interact with the services and responses received by honeypots. A low interaction honeypot is capable of emulating services, supporting network protocols and the main basic features real operating system possesses. It provides an attacker just about the bare minimum amount of information to make them interested in performing an attack and their actions will be logged. Nonetheless, as Naik and Jenkins [18] discuss, they provide no defence against spoofing attacks, as opposed to higher interaction levels, due to the limited capability of the responses the honeypot can return.

A medium interaction honeypot combines the strongest aspects of both the low and high interaction counterparts. Through this, it could gain a better grasp of attacks by offering slightly more interaction through responses and level of service emulation than low level interaction honeypots. The difference between medium and high interaction is the risk level associated. This type of honeypot runs on the virtual application layer and is therefore not fully emulating an operational system environment. Instead it emulates an application layer so that it logs connections, like low level interaction honeypots, but also offers more interaction. For instance, it can go as far as downloading any malware sample without the risk of affecting the rest of the system [19].

Finally, a high interaction honeypot offers the most realistic infrastructure. It is composed of a real computer system, providing real services and operating systems, and is setup in a way that it is extremely like the organisation’s infrastructure. It provides the most accurate data that is captured as attackers will assume to have direct access to a productive system, which makes it hard to discover they are interacting with a honeypot [20].

### 2.3. Related Work

In 2012, Brown et al. [21] conducted a study focusing primarily on Microsoft Azure and Amazon Web Service (AWS) instances. They deployed instances in servers all over the world, specifically including 22 on AWS and 14 on Azure. This work used some of these honeypots, namely Dionaea, Kippo (now Cowrie), Glastopf and Honeyd. Their results showed a large percentage of attacks originating from China, United States and Russia, similarly to our results. Nonetheless our results suggest that at the time that our experiments were conducted, Vietnam and India had a significantly larger percentage of occurrence as the visible attack origin. SSH attacks on the Kippo & Cowrie honeypots show similar usernames and passwords being used. Nonetheless in our results a wider distribution of different credentials exists, instead of username *root* and password *123456* dominating, as in [21]. Moreover, this study reported similar SSH input commands, with commands appearing to carve CPU information alongside any kind of details regarding the computer’s hardware appearing to be one of the focuses. The operating systems used by the attackers are also similar to our results. In AWS around 30% of attacks originated from Linux distributions followed by around 30% from Windows. The Windows machines were dominantly Windows 2000 SP4, which was the dominant OS at the time. Azure returned almost 83% of the attacks as unknown operating systems.What is perhaps most interesting about this study is the number of attacks was significantly less in total. China, the country with the most attacks on AWS in [21], contributed less than 2000 attacks. In comparison in our work more than 10 K attacks against our AWS instance originated from China.

Sophos deployed SSH honeypots in servers all over the world using AWS [22]. Their aim was to ensure their experiment was setup with no affiliation to Sophos, or any other company, except the hosting provider. They found that 95.4% of the traffic captured in their honeypots originated from China.The average number of attacks is roughly 757 per hour, which is similar to our findings. They also report an enormous number of the attempted SSH logins that used default credentials, with *root* and password *123456* being the most popular pair. Finally, they also measured the time taken for the first attack to take place, with the shortest time being against Sao Paulo’s server (52 s) and the longest being Ireland’s server (1 h 45 min). This variation was not found in our work as each server’s first attack happened in less than 5 mins after it went live.

Chapendama [23] also used T-Pot software but over a two-week period in the EU region using Google Cloud Platform. Cowrie was the most attacked honeypot with 102 k connections in the 14-day span (35% originated from China), but more interestingly, Dionaea was not attacked at all.

Saadi and Chaoui [24], used a range of honeypots to study the behaviour of attackers and then use collected data to improve the performances of their IDS. They collected the attack types from IDS and honeypots data, finding that around 69% of the traffic was classed as abnormal and just 31% falling into the normal traffic category. They suggest that the addition of the honeypot and IDS in their cloud infrastructure increased the accuracy of the IDS, in turn reducing the rate of false negatives and positives.

Sochor and Zuzcak [25] used not only cloud servers but also honeypots deployed on a University and Secondary school network over a three-month period. These servers were deployed in the Czech Republic, Prague, and the Slovak Republic. Their results suggest that threat actors attempted to bypass authentication with the same credential combinations, with username *root* or *admin* and password *123456*. The top attacking country against their SSH honeypot was India which was targeted 8 more than the 2nd most targeted country, i.e., China. Russia followed closely in 3rd place. Finally, in their experiments India was found leading the attacks.

Our work differentiates from past literature, which includes works that are limited to specific honeypots, services or protocols, without providing a holistic approach. Brown et al. [21] used Azure and AWS, which is only two of the prominent cloud providers and focused primarily on SSH sessions. On the contrary our work included 9 different honeypots, covering almost all services available, and used also an IDS. Other works used a much different methodology, such as the work by Wählisch et al. [26], involving using Android and iOS environments to analyse threats through honeypots. Others include deploying honeypots on University networks, like Sochor and Zuzcak [25]. Finally, compared to Bove et al. [27] our work uses far more honeypots deployed in all the popular cloud providers, which are operating in more regions. This allowed us to maximise the data that can be collected with our honeypots providing us with the regional differences of the threat actors’ activity during our data capture.

## 3. Methodology

Our work is based on the combination of IaaS and containers. Specifically, docker containers were deployed on Google Cloud, AWS, and Microsoft Azure with the use of T-Pot [28]. For our experiments we used a combination of low and medium interaction honeypots, which are commonly used by the literature, as they require less resources. Even though high interaction honeypots provide a more realistic environment, thus capturing more data from the attacker, they introduce more risks to the testing environment, as well as require more resources. As such, considering the operational costs for the duration of our experiments, we used the docker images of the following tools for data collection and data analysis, namely:

- Dionaea [29]. A low-interaction honeypot, emulates HTTP, FTP, TFTP, SMB, MSSQL and VOIP, with support for TLS and IPv6. Logging goes beyond straightforward service logs, as this work intends to capture actual malware payloads, with support for shellcode detection.
- Cowrie [30]. Is a medium-interaction honeypot which logs all forms of brute-force attacks and the preceding shell commands performed via SSH and Telnet. This tool can mimic successful compromises and can then log further interactions, which may be useful in uncovering an attacker’s motivations [31].
- Glutton [32]. A low-interaction honeypot which captures, logs and analyses traffic, supporting TCP and SSH. Glutton listens on all ports and takes action based on a rule file, allowing for fake SSH session interaction, which closes the connection after accepting a username and password.
- Heralding [33]. A low-interaction honeypot which emulates several login interfaces to capture entered credentials, as well as source/destination ports. Supports a variety of protocols, which includes, but is not exclusive to, Telnet, SSH, FTP, HTTP, POP and SMPT.
- Glastopf [34]. A low-interaction honeypot which emulates a very vulnerable Web server, hosting a variety of pages and applications. Glastopf utilises both Remote File Inclusion (RFI) and Local File Inclusion (LFI) techniques, extracting strings which allow it to emulate expected responses and lead an attacker to believe it was exploited successfully.
- Mailoney [35]. A low-interaction SMTP honeypot, which logs all emails and credentials, as well as exploit commands.
- VNClowpot [36]. A low-interaction Remote Desktop Protocol (RDP)/VNC honeypot. It logs attempted connections, responses and authentication attempts [34].
- RDPY [37]. A low-interaction RDP honeypot, implementing the Microsoft RDP protocol in Python. Collects login data, but it also capable of recording the actual RDP session.
- Honeytrap [38]. Acts as a catch-all for any traffic which has not been captured by another honeypot in T-pot. It monitors incoming packets and spawns TCP and UDP listeners, allowing for traffic to be recorded.
- Suricata [39]. A real-time Intrusion Detection System (IDS), which offers network security monitor functionality and has in-line Intrusion Prevention Systems (IPS). Suricata uses both rule-sets and signatures and supports detection of complex threats [40].

One of the aims of this work is to examine whether differences exist in the attackers’ activity when: (a) different honeypots are deployed on different cloud providers and (b) these honeypots are operating on different IaaS geographical regions. As a result, the containers were configured on the different IaaS instances and deployed in both the same geographical region and a combination of different locations (Table 1). This allowed, not only a direct comparison can be achieved through the instances being deployed on a server in the same area, but also a direct comparison of attack demographics across different regions. The instances were deployed 24 h per day for a total of 3 weeks during May 2020. Each started and ended at the same time to mitigate differences due to week-to-week variations in the threat landscape. The data that were collected via the honeypots were further enriched with contextual information, such as geolocation providers and threat intelligence feeds (based on open source blacklisting providers). Moreover, the P0f tool used for passive traffic fingerprinting in order to identify the operating system of the IP source.

Cloud infrastructure providers allow the choice of server geographical location and IP address range that will be allocated. As depicted in Table 1 the first deployment of the cloud instances used were in the East region of the United States. This also tends to be the default and cheapest server region to be offered by most cloud providers. This region was chosen as it attracts many customers [41], suggesting that there may be demographic difference between the companies choosing their host regions, which may be reflected in the attacker demographics and intentions. The region chosen for AWS was the *us-east-1c*, which is based in North Virginia in the East region of the United States. Similarly, for Google Cloud the *us-east-4c* region was used, which is based in Ashburn, North Virginia. Finally, for Microsoft Azure the region *East US* was used, which is also based in Virginia.

The second set of servers used different regions all over the world. This was to allow a direct comparison of attack demographics across 3 regions. The 3 regions chosen were North America, Europe, and Asia. More specifically, (i) for AWS server the same area was reused, i.e., North America continent, (ii) for Google Cloud the region *europe-west2-c* was used, and (iii) for Microsoft Azure the region *Southeast Asia* was used.

Each instance had Debian 9 (Stretch) as the operating system and was equipped with 2 virtual CPUs, 6 GB of memory and a 60 GB hard drive for the duration of the project, i.e., 3 weeks. This was adequate to store all the logs from the honeypots for that period and helped ensure operational costs were kept low to maintain the budget allocated. The actual configuration parameters for each instance are provided in Table 2, with firewall settings provided in Table 3.

The honeypots expose a range of both popular and less popular ports. These include SSH (on port 22) and HTTP/HTTPS (on port 80 and 443) and therefore cannot be used to access the main dashboard, provided by T-Pot. Hence, port 64297 was used to provide secure access to the Dashboard over HTTPS and port 64295 for SSH access.

As a result, we allowed access to port range 0-64000 for all source IP addresses (i.e., the entire Internet), which allows access to all the different honeypots. Any port above 64000, was reserved for the management of our test infrastructure and for the data analysis. The data collected from the honeypots were logged with Logstash and were analysed and visualised with Elasticsearch and Kibana, which are all part of Elastic Stack. Elastic Search is built upon Apache Lucene and is released with an Apache license. It uses the architectural style REST (REpresentational State Transfer) for its distributed search engine. Kibana, works with elastic search and data visualisation and analytics-based software package using the data input from the other members of the elastic stack. Finally, Logstash is used as a data collection engine that makes the storage and collection of log files easy and quick to implement.

## 4. Results

This section discusses the results based on the analysis of the data that were collected in (a) the US region and (b) the Europe, Asia and US regions.

### 4.1. Deployment in East US Region

This subsection provides an analysis of the logs that were collected by the honeypots that were deployed on (a) AWS, (b) GC, and (c) Azure cloud providers. The analysis of the logs that were generated over the three-week period (in May 2020) uncovered: (a) 248,144 attacks in the AWS honeypots (b) 581,116 attacks in the GC honeypots, and (c) 340,735 attacks in the Azure honeypots. Amongst the honeypots that we deployed to the cloud providers, Dionaea, Cowrie and Glutton identified most of the attacks.

#### 4.1.1. Amazon Web Services (AWS)

**Figure 1:**
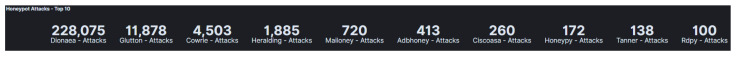
AWS Honeypot Attacks—Top 10.

**Figure 2:**
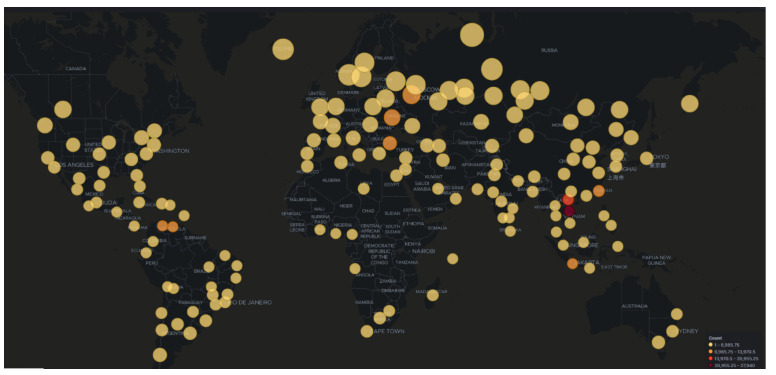
AWS Honeypot Attack Map.

As illustrated in Figure 1 Dionaea was the most targeted honeypot accounting for 91% of the total attacks, followed by Glutton (5% of attacks) and Cowrie (2% of attacks). Further inspection of the collected data revealed that there were over ten countries involved in the attacks, the majority of them residing in America, Asia, and Europe (Figure 2). Amongst them Vietnam seems to host the highest volume (30%—47,510 attacks) of total attacks, followed by Russia and Venezuela. It is worth noting that these data capture the visible attack origin. This holds true as with technologies such as VPNs available or based on the fact that a threat actor could use other compromised systems as stepping stones, these data may not be an accurate representation of the attack’s origin.

**Figure 3:**
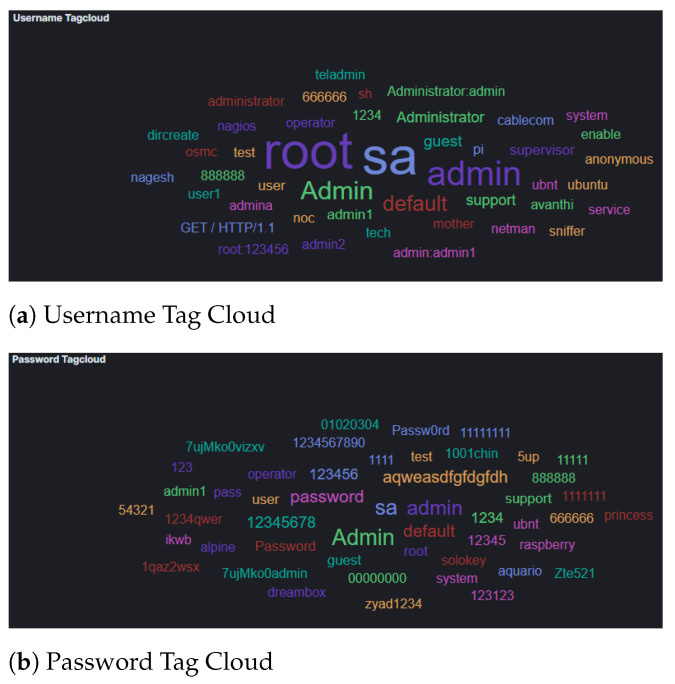
AWS Cowrie Username and Password Tag Cloud.

The results from Cowrie honeypot suggest that attackers used automation as well as default credentials and passwords found in a dictionary in order to to bypass authentication (Figure 3). Furthermore, the analysis of attacker input command captured by this honeypot indicated attempts to elevate their privileges and to perform (system) information gathering before attempting to fulfil their objectives [42]. Of interest however was the */bin/busybox CORONA*, which suggests that the attackers tried to use the honeypots for social engineering, such as spreading phishing emails or fake corona virus maps. It is worth noting that since our experiments took place during COVID-19 pandemic, this is an unsurprising tactic to be used by attackers. Furthermore, the use of busy box suggests that the attackers considered that they were interacting with IoT devices, which could be later used to host such social engineering attacks.

**Figure 4:**
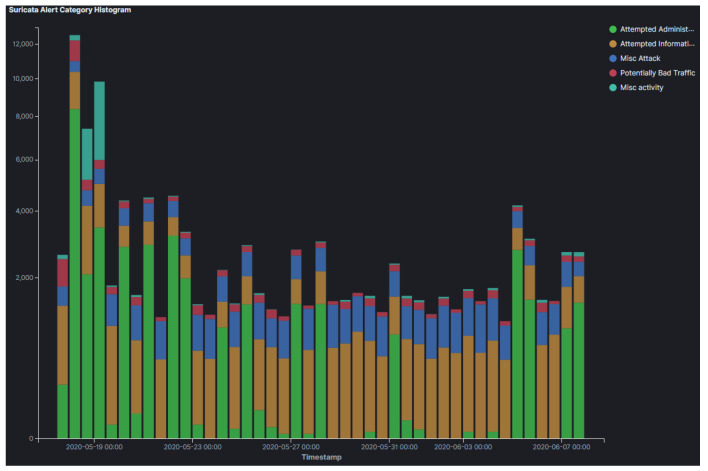
AWS Suricata Alert Category Histogram.

To achieve the aforementioned, based on the results that were identified from Suricata (Figure 4), the attackers tried to exploit various known vulnerabilities released in 2020 (i.e., the same year we run our experiments) going back up to 1999. The top targeted vulnerabilities include CVE-2006-2369, a vulnerability for the RealVNC software which allows attackers to bypass VNC authentication.

#### 4.1.2. Google Cloud

**Figure 5:**
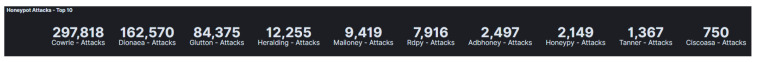
GCP Honeypot Attacks—Top 10.

The results from the Google Cloud instance were expected to be similar with the ones from the AWS instance, as they were both deployed in the same region, as well as data collection took place with the same honeypot configuration and at the same time period. Nonetheless, our analysis suggests discrepancies in the attackers’ activities, as summarized in (Figure 5). Firstly, while Dionaea honeypot logged most attacks on the AWS instance, Cowrie honeypot received most attacks on Google Cloud (51%—297,818). Specifically, Dionaea had significantly less attacks in this instance, namely 28% on Google Cloud vs. 91% on AWS. The Google Cloud server received far more attacks overall, with most honeypots receiving 10–20 times more attacks on Google Cloud than on AWS.

**Figure 6:**
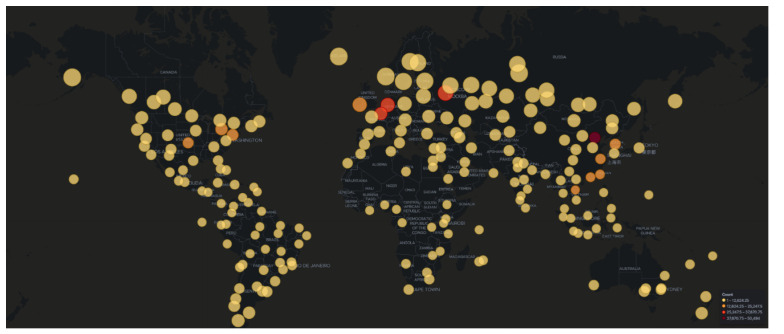
GCP Honeypot Attack Map.

The analysis of attacker source IPs produced different results in comparison to AWS honeypots, which align more with results from past research [27]. Firstly, China ranked first (over 25% of the attacks), followed by the United States (20% of attacks) who were not even included in the top 10 for AWS honeypots. Furthermore, Vietnam, which ranked first in AWS honeypots, ranked fourth after Russia. As depicted in the attack map in Figure 6, America, Asia and Europe dominate the results of the geolocation of the attacker’s source IP. Nonetheless, in this cloud provider the contribution of East Coast of the United States, where our honeypots were also deployed, is significantly higher compared to the results from AWS.

**Figure 7:**
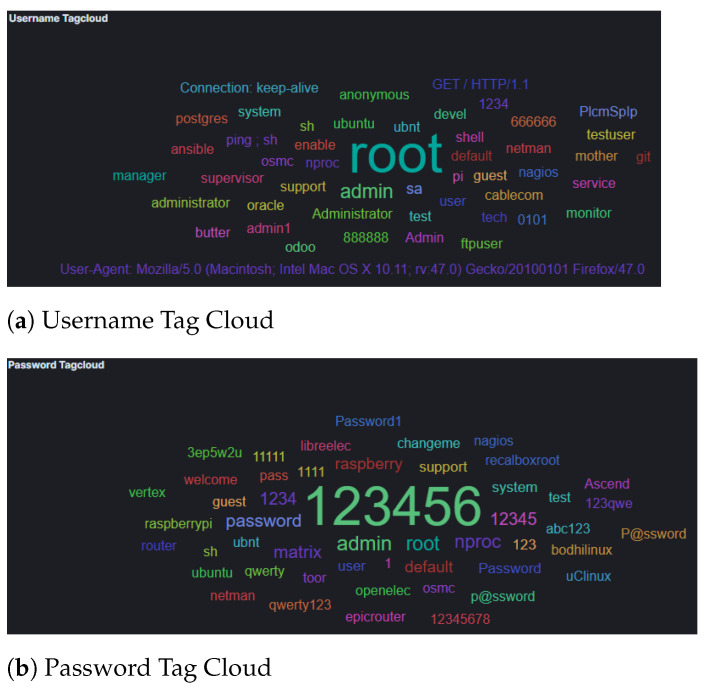
GCP Cowrie Username and Password Tag Cloud.

Analysis of the commands used, as they were collected by the Cowrie honeypot, suggest that the attackers used default credentials (username and passwords), as summarized in Figure 7. However, these data herein could indicate that some automated attack tools were used that were malfunctioning. This holds true as data collected in the username field included GET commands and User-Agent strings. Furthermore, our results suggest that, similar to the attackers’ activity in AWS, considerably more activity concentrated on (system) information gathering, where the attackers’ attempt to collect information about the CPU architecture and/or scheduled tasks (e.g., with commands such as *crontab, cat /proc/cpuinfo, freeem -m, etc.*), as well as privilege escalation.

**Figure 8:**
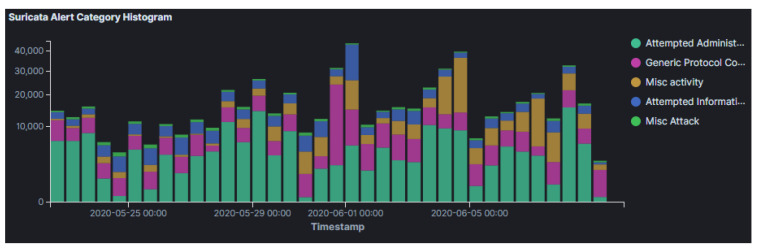
GCP Suricata Alert Category Histogram.

The analysis of the results from Suricata indicates the type of vulnerabilities that the attackers targeted, which are summarized in Figure 8. Our results herein suggest that the attackers attempted to exploit old versions of operating systems. Moreover, most of the targeted vulnerabilities that have been identified are considerably older in this cloud provider. The most targeted vulnerability was CVE-2001-0540, which is a memory leak in terminal servers running Windows 2000 and Windows NT, allowing remote attackers to cause a Denial of Service via purposely malformed RDP packets. This suggests that threat actors that targeted our honeypots were attempting to impair the security of extremely outdated operating systems, which might still be in use all over the world.

#### 4.1.3. Microsoft Azure

**Figure 9:**
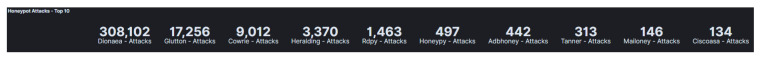
Microsoft Azure Honeypot Attacks.

**Figure 10:**
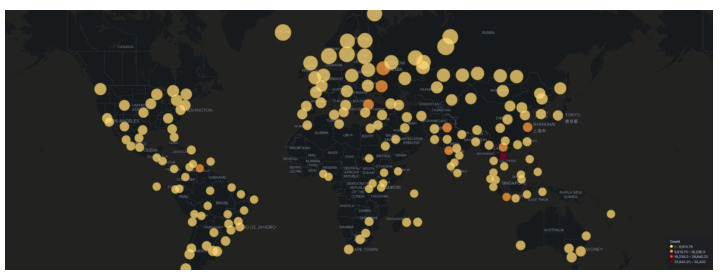
Microsoft Azure Honeypot Attack Map.

**Figure 11:**
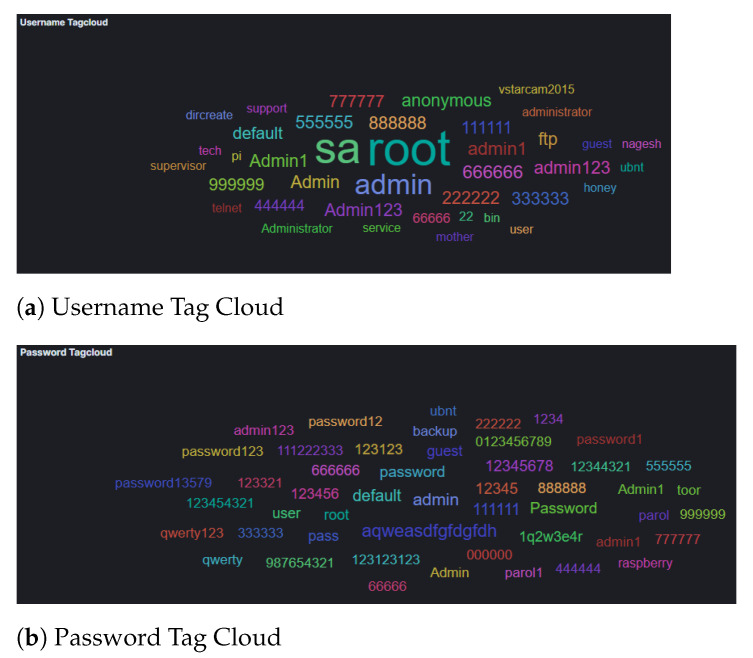
Microsoft Azure Cowrie Username and Password Tag Cloud.

**Figure 12:**
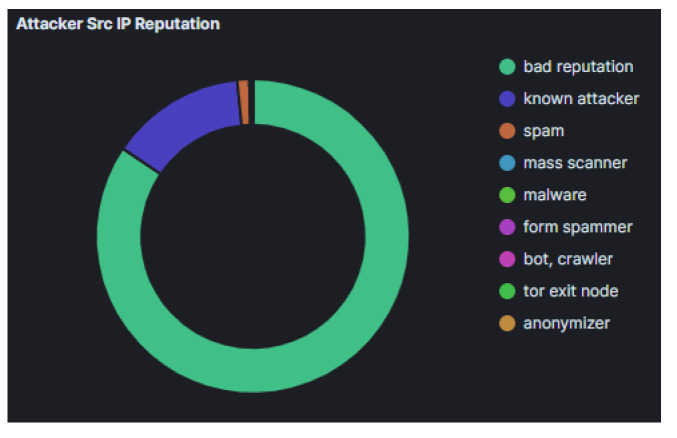
Microsoft Azure Attacker Src IP Reputation.

Attacks which were identified in the Azure honeypots were similar to those in AWS, with regards to their frequency (Figure 9) and geolocation (Figure 10). Specifically, the honeypots Dionaea, Cowrie and Glutton identified the majority of attacks that seems to originate from Vietnam (just over 25% of attacks), with Russia following. In addition, the analysis of the attackers’ activity suggests, similarly to the other two cloud providers, attempts to bypass authentication with automation by using common password lists (Figure 11), as well as to perform information gathering and privilege escalation (IPs are also tagged having bad reputation as illustrated in Figure 12). It is worth mentioning, that the commands that the threat actors used were almost identical to the AWS server. This could suggest that the same threat actors or software was used against these two cloud providers.

Moreover, similarities exist in the vulnerabilities that the attackers targeted. Specifically, CVE-2006-2369 was found 3371 times compared to 1924 in AWS. CVE-2001-0540 was found 1894 times in Azure and 150 times in AWS. These two vulnerabilities were found in GCP, as mentioned earlier, but more frequently (i.e., summative frequency of ∼27,000 compared to ∼5200 in Azure). CVE-2005-4050 followed in the ranking, it was captured 502 times on Azure but none in AWS.

#### 4.1.4. Overall Results

**Figure 13:**
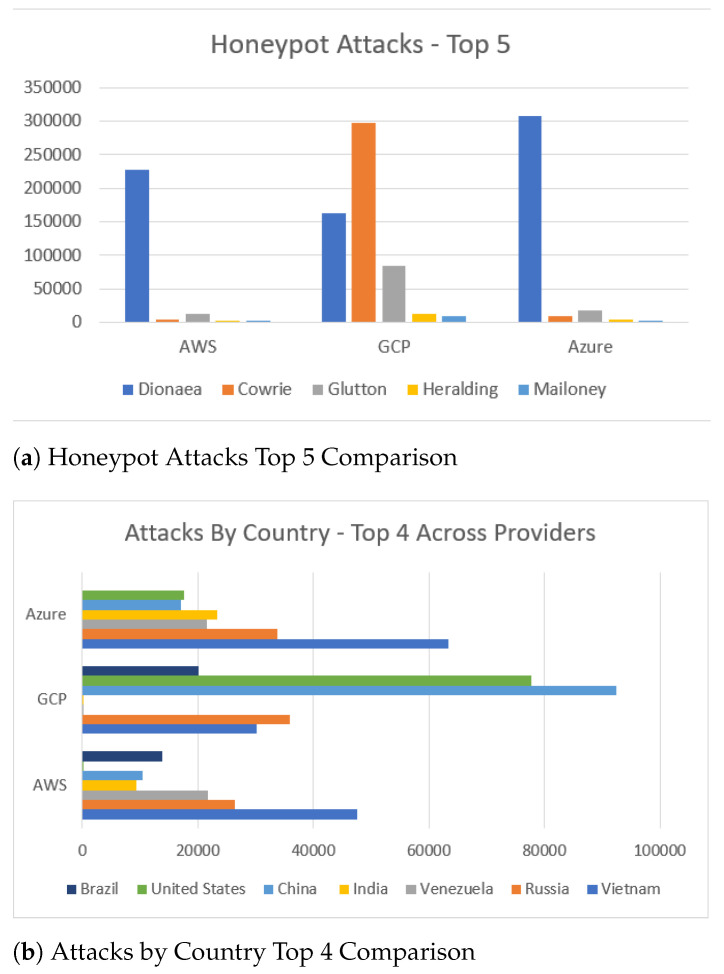
Top Honeypot Attacks Furthermore, Countries.

In total, the honeypots identified 1,169,995 attacks, i.e., over 55K attacks per day. One should note that the volume of attacks that were active at the time of our experiments could have been even greater as attacks maybe have been: (i) missed by the honeypots and (ii) aborted after an initial reconnaissance phase [43]. Moreover, since the honeypots were deployed in the regions and with the same configuration one would expect that they would capture similar data of attackers’ activity. Nonetheless, our results disprove this assumption and Figure 13a illustrates the disparities in attacks, both in their volume and the most targeted honeypots.

Overall, Dionaea, Cowrie and Glutton were the honeypots that received most of the attacks during our experiments. This is somewhat expected as they were hosting popular services that are often targeted by attackers, such as Web, SSH and Telnet. Our results also uncover instances of large volumes of attacks targeting SSH and Telnet services, which appear across a variety of ports numbers and not just their default port numbers. However, one should note the volume of data that has been collected by Dionaea, a honeypot that captures attackers’ shellcode, which emphasizes the prevalence of malware and payloads circulating the Internet and targeting its users.

Our results disprove the assumption that attackers would target the services on cloud providers based on the cloud providers’ market share. This holds true, as the most attackers’ activity was identified by the honeypots that were deployed on Google Cloud, which at the time of experiments had the smallest market share amongst the cloud providers [44]. With regards to attackers’ geolocation (Figure 13b), the majority of attacks captured by the AWS and Azure honeypots originated from Vietnam and Russia, whereas in the Google Cloud honeypots around half of the attacks seemed to originate from China and the United States.

**Figure 14:**
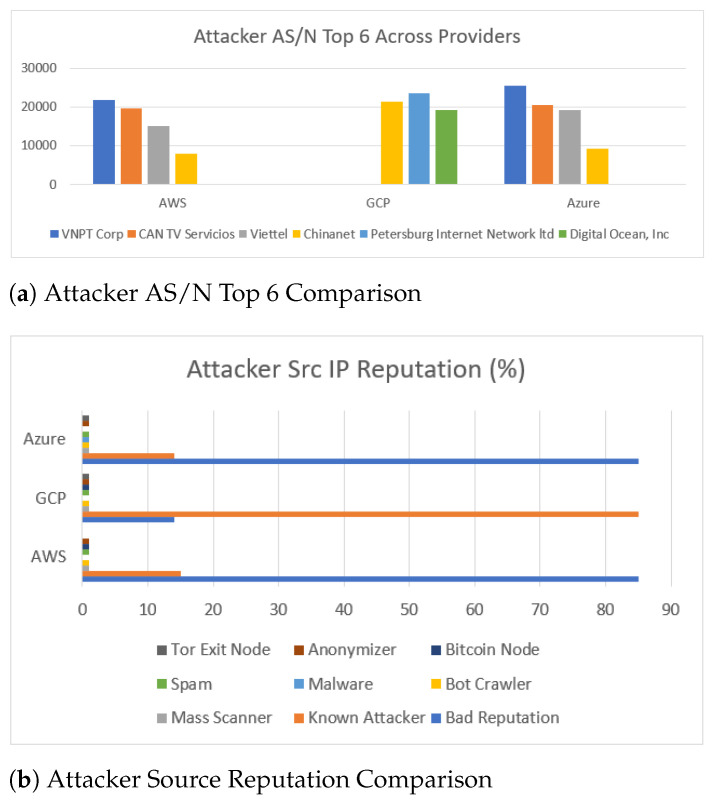
Attacker AS/N Furthermore, Attacker Source Reputation Comparisons.

Nonetheless, these geolocations indicate the last node that accessed our service, e.g., in case of the use of stepping stones or VPN technology, therefore it is infeasible to use this information in order to infer the motives of the attackers. A further analysis of the ISP of these nodes, is summarized in Figure 14a, showing that the same ISP providers appear across the three cloud servers. These include providers, such as Digital Ocean, which are known to host IP addresses that are malicious or flagged with bad reputation. This suggests that an organization is able to prevent these nodes from accessing their online services by filtering of the source IPs.

This is evident in Figure 14b, which summarizes the reputation of the source IP address of the attacks that we have identified. The majority of source IPs come from sources with a known bad reputation, which suggests that they can be filtered out by an organisation that keeps a combination of up-to-date threat intelligence feeds [45]. Specifically, in the cases of AWS and Azure, these IPs are known to have *bad reputation* and constitute 80% of the source IPs that were collected. The remainder of source IPs collected includes the *known attackers* category (∼15% of source IPs), with the rest of the threat intelligence categories, such as spam, being less than 5% of the source IPs. On the contrary in GCP these results are reversed, namely over 80% of source IPs are classified to *known attacker* category and only ∼15% in *bad reputation*.

Our results from Suricata highlight two vulnerabilities against graphical desktop sharing software, namely CVE-2006-2369 and CVE-2001-0540, as the most targeted vulnerabilities which were found in all cloud providers. CVE-2006-2369, a vulnerability in the RealVNC software, was the most frequent vulnerability targeted in AWS and Azure. CVE-2001-0540, a vulnerability in the RDP software, was the most targeted vulnerability in GCP. While uncovering that attackers attempted to exploit remote sharing software in order to get unauthorized access to these devices is not surprising, the fact that this vulnerability was the most sought after during the pandemic when most organisations switched to remote working increases the impact of this finding. In addition, in many cases attackers attempted to exploit vulnerabilities that were considerably old, which highlights the importance of rigorous patch management.

**Figure 15:**
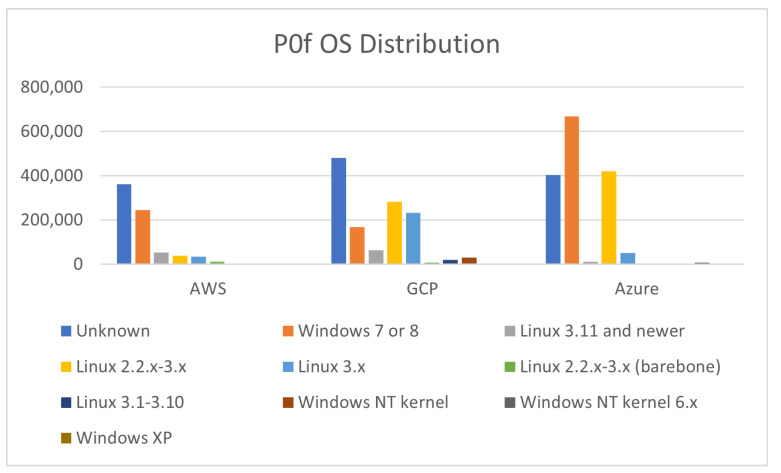
OS Distribution Comparison.

Finally, Figure 15 summarizes the operating systems of the devices that communicated with our honeypots. Our results indicate that attacking devices were running older versions of Microsoft Windows, including 7 or 8, and XP without any occurrence of Windows 10. However, the majority of the attacking OS included various Linux kernels, which was expected as many Linux distributions exist preloaded with tools that can be used to exploit software or systems. Furthermore, Linux as well as older Windows operating systems, have minimal resource requirements, making them relatively inexpensive options for deploying at scale on cloud platforms as part of an attack campaign.

### 4.2. Deployment in Different Regions

**Figure 16:**
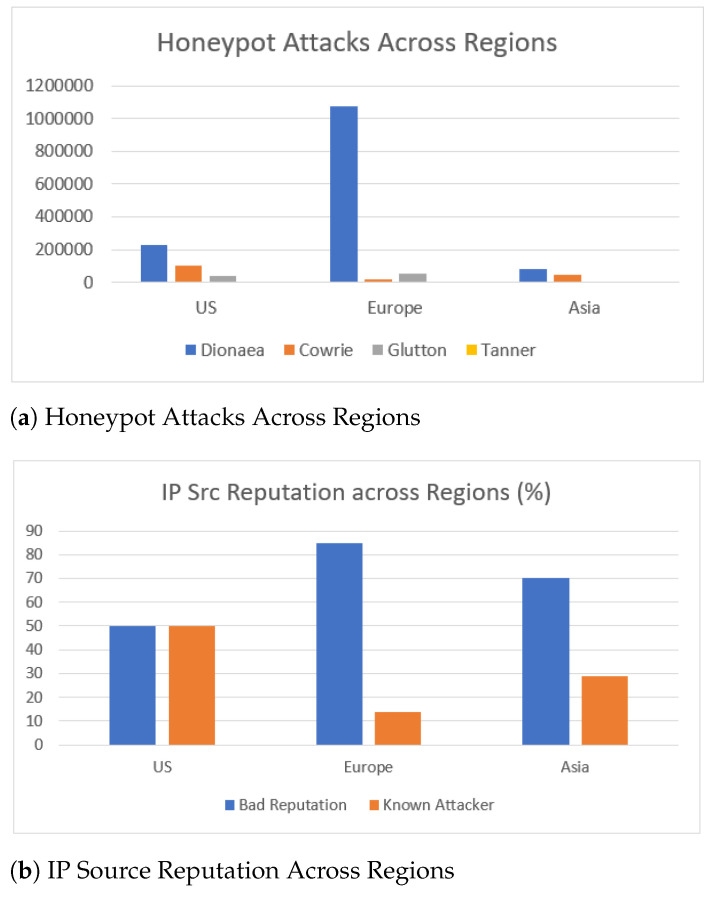
Honeypot Attacks and IP Source Reputation Across Regions.

Figure 16a summarizes the frequency of attacks in the most targeted honeypots across the three regions. Dionaea honeypot deployed on GCP in the European region, received the most attacks, namely more than 1 million attacks. On the US and Asia region, SSH exploits were more prominent as suggested by the data captured by Cowrie honeypot. These attacks were infrequent in Europe, as depicted in Figure 16a. This finding is similar to those of past research [27].

Figure 16b summarizes the threat intelligence information for the source IPs that accessed our honeypots in the three regions. These suggest that the majority of the source IPs originate from Europe and Asia were classified as *bad reputation*, whereas the classes *bad reputation* and *known attacker* evenly occurred in the US region.

**Figure 17:**
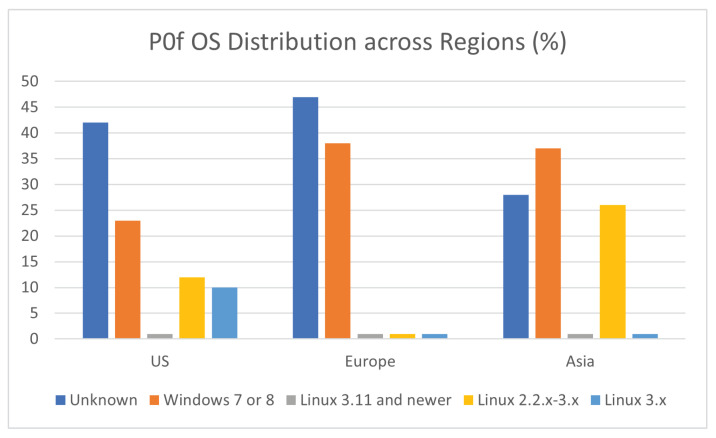
OS Distribution Across Regions.

With regards to the operating systems of the attacking devices, the results are summarized in Figure 17. Each region had many devices with unknown operating systems, which suggests that most times the attackers were circumventing the fingerprinting activities of Pof. The Asia region was the only region where an identified OS, i.e., Windows 7 or 8, was more frequent than this unrecognised class. It is worth noting that our results suggest that Windows was the most frequently used operating system. This based on the fact that the majority of vulnerabilities targeted remote desktop sharing software, might suggest that the attacker aimed to simulate the traffic from normal users. We also assume that some of these devices may be part of a botnet without the knowledge and consent of their owner. Linux 2.2.x–3.x was found more often in Asia, was rarely found in Europe and was the third most common OS in the US. Newer Linux versions (i.e., version 3.11 and newer) were hardly found across all regions, with Linux 3.x being more frequently encountered in the US region.

Similarly to what was noticed in the honeypots that were only deployed in the US Region, our results suggest that threat actors targeted vulnerabilities against software which became very popular during the time of our experiments due to the pandemic. In these experiments these included remote desktop sharing and VoIP. Amongst them the most targeted vulnerabilities included: *(i)* CVE-2006-2369 (RealVNC) in the US region, *(ii)* CVE-2001-0540 (RDP) in EU and Asia regions, *(iii)* CVE-2012-0152 (RDP) in the Asia region, and *(iv)* CVE-2005-4050 (VoIP) in EU region. Finally, it is worth noting that these vulnerabilities were not recently uncovered, but they dated as early as 2001.

## 5. Conclusions

The ability to adapt to any workload changes with the automatic provisioning and deprovisioning of resources, has made cloud computing necessary for the operation of every organisation and especially SMEs. However, the increasing popularity of cloud infrastructures did not come without risk, as it has attracted the attention of threat actors. In this work honeypots were deployed on all popular cloud providers, operating in multiple regions, i.e., North America, Europe and Asia, in order to study the techniques employed by the threat actors. Our analysis shows the regional differences of the threat actors’ activity during our data capture, which coincided with the first wave of the COVID-19 pandemic. We find evidence of automated activity, which targets popular protocols, such as remote desktop sharing, which became prevalent during the pandemic. Our results suggest that the threat actors, targeted our assets irrespective of the popularity of the cloud provider that they were deployed, as the majority of the attacks were captured by the honeypots that were deployed on Google Cloud, i.e., the provider with the lowest market share during our experiments. In addition, our analysis provides interesting findings with regards to the provenance of the attacks. Specifically, our results suggest that currently attackers often use exit nodes originating, not only from countries that are considered common sources of attacks, e.g., China, Russia and USA, but also uncommon sources, such as Venezuela, India, Brazil and Vietnam.

Our results, while constrained by the duration of our experiments, highlight the importance of facilitating rigorous patch management and threat intelligence feeds for an organisation that operates online, irrespective of whether a cloud provider is used. Moreover, our results provide insights on adversarial activity, during our data collection, which can be used to inform the Situational Awareness operations of an organisation. Our plans for future work, include data collection for longer periods of time, which can provide insights of threat actors’ activity over time. Furthermore, we plan to further investigate the collected logs, combining manual analysis, in order to identify instances of unknown attacks or use of zero-day vulnerabilities.

## Figures and Tables

**Table 1:** Server Provider and Region Per Deployment.

Provider	Deployment	Server Region	Actual Location
Google Cloud	1	us-east-4c	Ashburn, North Virginia, US
Google Cloud	2	europe-west2-c	London, England, UK
Amazon (AWS)	1	us-east-1c	North Virginia, US
Amazon (AWS)	2	us-east-1c	North Virginia, US
Microsoft Azure	1	East US	Virginia, US
Microsoft Azure	2	Southeast Asia	Singapore, Malaysia

**Table 2:** GCP, AWS and Azure Instance Specifications.

Cloud Prov.	Machine Type	CPU Count	RAM	Storage	Server Zone
Google Cloud	n1-standard-2	2	7.5 GB	60 GB SSD	europe-west2-c
Google Cloud	n1-standard-2	2	7.5 GB	60 GB SSD	us-east4-c
AWS	t2.large	2	8 GB	60 GB SSD	us-east-1c
Microsoft Azure	Standard D2s v3	2	8 GB	60 GB SSD	Southeast Asia
Microsoft Azure	Standard D2s v3	2	8 GB	60 GB SSD	East US

**Table 3:** Firewall Rules for All Instances.

Name	Type	Filters	Protocols /Ports	Action
**Allow Private Connection**	Ingress	Private IP Address	64295-64297	Allow
**Attack Traffic**	Ingress	IP ranges 0.0.0.0/24	0-64000	Allow

## References

[1] Fadilpašić S. (2020). One in Four Companies Will Be All-Cloud within a Year. https://www.itproportal.com/news/one-in-four-companies-will-be-all-cloud-within-a-year/.

[2] Villas-Boas A. (2020). Security Researchers Warned for Years about the Cloud-Security Flaw Used in the Massive Capital One Hack, However, Amazon Apparently Leaves It up to Customers to Protect. https://www.businessinsider.com/capital-one-hack-vulnerability-on-cloud-amazon-known-for-years-2019-8.

[3] Moore C., Al-Nemrat A. (2015). An analysis of honeypot programs and the attack data collected. Proceedings of the International Conference on Global Security, Safety, and Sustainability.

[4] Sharma A. (2013). Honeypots in Network Security. Int. J. Technol. Res. Appl..

[5] Hall A.J., Pitropakis N., Buchanan W.J., Moradpoor N. Predicting malicious insider threat scenarios using organizational data and a heterogeneous stack-classifier. Proceedings of the 2018 IEEE International Conference on Big Data (Big Data).

[6] Kandias M., Mylonas A., Virvilis N., Theoharidou M., Gritzalis D. (2010). An insider threat prediction model. Proceedings of the International Conference on Trust, Privacy and Security in Digital Business.

[7] Shendre K., Sahu S.K., Dash R., Jena S.K. (2016). Learning probe attack patterns with Honeypots. Proceedings of the 3rd International Conference on Advanced Computing, Networking and Informatics.

[8] Slahor S. (2011). What is cloud computing. ProQuest Educ. J..

[9] Liu F., Tong J., Mao J., Bohn R., Messina J., Badger L., Leaf D. (2011). NIST cloud computing reference architecture. NIST Spec. Publ..

[10] Pitropakis N., Darra E., Vrakas N., Lambrinoudakis C. It’s All in the Cloud: Reviewing Cloud Security. Proceedings of the 2013 IEEE 10th International Conference on Ubiquitous Intelligence and Computing and 2013 IEEE 10th International Conference on Autonomic and Trusted Computing.

[11] Stoll C. (2005). The Cuckoo’s Egg: Tracking a Spy through the Maze of Computer Espionage.

[12] Cheswick B. An Evening with Berferd in which a cracker is Lured, Endured, and Studied. Proceedings of the Winter USENIX Conference.

[13] Spitzner L. (2003). The honeynet project: Trapping the hackers. IEEE Secur. Priv..

[14] Canner B. (2020). The Cybersecurity Honeypot: What You Need to Know. https://solutionsreview.com/security-information-event-management/cybersecurity-honeypot-need-know/.

[15] Spitzner L. (2001). The value of honeypots, part one: Definitions and values of honeypots. Secur. Focus.

[16] Pitropakis N., Panaousis E., Giannakoulias A., Kalpakis G., Rodriguez R.D., Sarigiannidis P. (2018). An enhanced cyber attack attribution framework. Proceedings of the International Conference on Trust and Privacy in Digital Business.

[17] Chacon J., McKeown S., Macfarlane R. Towards Identifying Human Actions, Intent, and Severity of APT Attacks Applying Deception Techniques—An Experiment. Proceedings of the 2020 International Conference on Cyber Security and Protection of Digital Services (Cyber Security).

[18] Naik N., Jenkins P. A fuzzy approach for detecting and defending against spoofing attacks on low interaction honeypots. Proceedings of the 2018 21st International Conference on Information Fusion (Fusion).

[19] Zuzčák M., Zenka M. (2020). Expert system assessing threat level of attacks on a hybrid SSH honeynet. Comput. Security.

[20] Memari N., Hashim S., Samsudin K. (2015). Network probe patterns against a honeynet in Malaysia. Defences S&T Tech. Bull..

[21] Brown S., Lam R., Prasad S., Ramasubramanian S., Slauson J. (2012). Honeypots in the Cloud.

[22] Boddy M. (2020). Exposed: Cyberattacks on Cloud Honeypots. https://www.sophos.com/en-us/medialibrary/PDFs/Whitepaper/sophos-exposed-cyberattacks-on-cloud-honeypots-wp.pdf.

[23] Chapendama S. (2020). Analysing Honeypot Data Using Kibana and Elasticsearch. https://towardsdatascience.com/analysing-honeypot-data-using-kibana-and-elasticsearch-5e3d61eb2098.

[24] Saadi C., Chaoui H. (2016). Cloud computing security using IDS-AM-Clust, Honeyd, honeywall and Honeycomb. Procedia Comput. Sci..

[25] Sochor T., Zuzcak M. (2014). Study of internet threats and attack methods using honeypots and honeynets. Proceedings of the International Conference on Computer Networks.

[26] Wählisch M., Vorbach A., Keil C., Schönfelder J., Schmidt T.C., Schiller J.H. (2013). Design, implementation, and operation of a mobile honeypot. arXiv.

[27] Bove D., Müller T. Investigating characteristics of attacks on public cloud systems. Proceedings of the 2019 6th IEEE International Conference on Cyber Security and Cloud Computing (CSCloud)/2019 5th IEEE International Conference on Edge Computing and Scalable Cloud (EdgeCom).

[28] Telekom Security (2016). Github Telekom-Security/Tpotce. https://github.com/telekom-security/tpotce.

[29] Sethia V., Jeyasekar A. Malware Capturing and Analysis using Dionaea Honeypot. Proceedings of the 2019 International Carnahan Conference on Security Technology (ICCST).

[30] Michel Oosterhof (2015). GitHub—Cowrie/Cowrie: Cowrie SSH/Telnet Honeypot. http://cowrie.readthedocs.io.

[31] Deshmukh S., Rade R., Kazi D. (2019). Attacker Behaviour Profiling using Stochastic Ensemble of Hidden Markov Models. arXiv.

[32] MushMush Foundation (2016). Github Mushorg/Glutton. https://github.com/mushorg/glutton.

[33] Vestergaard J. (2012). Github Johnnykv/Heralding. https://github.com/johnnykv/heralding.

[34] Mphago B., Bagwasi O., Phofuetsile B., Hlomani H. Deception in dynamic web application honeypots: Case of glastopf. Proceedings of the International Conference on Security and Management (SAM), The Steering Committee of The World Congress in Computer Science, Computer.

[35] Awhitehatter (2015). Github Awhitehatter/Mailoney. https://github.com/awhitehatter/mailoney.

[36] McMurray J.S. (2016). Github Magisterquis/Vnclowpot. https://github.com/magisterquis/vnclowpot.

[37] Peyrefitte S. (2013). Github Citronneur/Rdpy. https://github.com/citronneur/rdpy.

[38] Werner T. (2020). Honeytrap-a Dynamic Meta-Honeypot Daemon. http://honeytrap.carnivore.it/documentation/.

[39] (2016). Github OISF/Suricata. https://github.com/OISF/suricata.

[40] Nam K., Kim K. A study on sdn security enhancement using open source ids/ips suricata. Proceedings of the 2018 International Conference on Information and Communication Technology Convergence (ICTC).

[41] Marquez E. (2020). Save Yourself a Lot of Pain (and Money) by Choosing Your AWS Region Wisely. https://www.concurrencylabs.com/blog/choose-your-aws-region-wisely/.

[42] Hutchins E.M., Cloppert M.J., Amin R.M. (2011). Intelligence-driven computer network defense informed by analysis of adversary campaigns and intrusion kill chains. Lead. Issues Inf. Warf. Secur. Res..

[43] Nisioti A., Mylonas A., Yoo P.D., Katos V. (2018). From intrusion detection to attacker attribution: A comprehensive survey of unsupervised methods. IEEE Commun. Surv. Tutor..

[44] Cloud Consulting Europe (2020). Cloud Computing—The Five Best Cloud Providers of 2020.

[45] Virvilis N., Mylonas A., Tsalis N., Gritzalis D. (2015). Security Busters: Web browser security vs. rogue sites. Comput. Secur..

